# Venous thromboembolism and bleeding in critically ill patients with severe renal insufficiency receiving dalteparin thromboprophylaxis: prevalence, incidence and risk factors

**DOI:** 10.1186/cc6810

**Published:** 2008-03-03

**Authors:** Deborah Cook, James Douketis, Maureen Meade, Gordon Guyatt, Nicole Zytaruk, John Granton, Yoanna Skrobik, Martin Albert, Robert Fowler, Paul Hebert, Guiseppe Pagliarello, Jan Friedrich, Andreas Freitag, Tim Karachi, Christian Rabbat, Diane Heels-Ansdell, William Geerts, Mark Crowther, the Canadian Critical Care Trials Group

**Affiliations:** 1 Department of Medicine, 1200 Main Street, McMaster University, Hamilton, Canada, L8N 3Z5; 2 Department of Clinical Epidemiology and Biostatistics, 1200 Main Street, McMaster University, Hamilton, Canada, L8N 3Z5; 3 Department of Medicine, University of Toronto, 190 Elizabeth Street, Toronto, Canada, M5G 2C4; 4 Interdepartmental Division of Critical Care Medicine, 190 Elizabeth Street, University of Toronto, Toronto, Canada, M5G 2C4; 5 Department of Medicine, Université de Montréal, Pavillon Roger-Gaudry 2900, boul. Édouard-Montpetit, Montréal, Canada, H3T 1J4; 6 Department of Critical Care, Université de Montréal, Pavillon Roger-Gaudry 2900, boul. Édouard-Montpetit, Montréal, Canada, H3T 1J4; 7 Department of Critical Care, University of Ottawa, 501 Smyth Road, Box 201, Ottawa, Canada, K1H 8L6

## Abstract

**Background:**

Critically ill patients with renal insufficiency are predisposed to both deep vein thrombosis (DVT) and bleeding. The objective of the present study was to evaluate the prevalence, incidence and predictors of DVT and the incidence of bleeding in intensive care unit (ICU) patients with estimated creatinine clearance <30 ml/min.

**Methods:**

In a multicenter, open-label, prospective cohort study of critically ill patients with severe acute or chronic renal insufficiency or dialysis receiving subcutaneous dalteparin 5,000 IU once daily, we estimated the prevalence of proximal DVT by screening compression venous ultrasound of the lower limbs within 48 hours of ICU admission. DVT incidence was assessed on twice-weekly ultrasound testing. We estimated the incidence of major and minor bleeding by daily clinical assessments. We used Cox proportional hazards regression to identify independent predictors of both DVT and major bleeding.

**Results:**

Of 156 patients with a mean (standard deviation) creatinine clearance of 18.9 (6.5) ml/min, 18 had DVT or pulmonary embolism within 48 hours of ICU admission, died or were discharged before ultrasound testing – leaving 138 evaluable patients who received at least one dose of dalteparin. The median duration of dalteparin administration was 7 days (interquartile range, 4 to 12 days). DVT developed in seven patients (5.1%; 95% confidence interval, 2.5 to 10.1). The only independent risk factor for DVT was an elevated baseline Acute Physiology and Chronic Health Evaluation II score (hazard ratio for 10-point increase, 2.25; 95% confidence interval, 1.03 to 4.91). Major bleeding developed in 10 patients (7.2%; 95% confidence interval, 4.0 to 12.8), all with trough anti-activated factor X levels ≤ 0.18 IU/ml. Independent risk factors for major bleeding were aspirin use (hazard ratio, 6.30; 95% confidence interval, 1.35 to 29.4) and a high International Normalized Ratio (hazard ratio for 0.5-unit increase, 1.68; 95% confidence interval, 1.07 to 2.66).

**Conclusion:**

In ICU patients with renal insufficiency, the incidence of DVT and major bleeding are considerable but appear related to patient comorbidities rather than to an inadequate or excessive anticoagulant from thromboprophylaxis with dalteparin.

**Clinical Trial Registration:**

Number NCT00138099.

## Introduction

Approximately 30% of critically ill patients admitted to an intensive care unit (ICU) will have renal insufficiency at the time of admission or will develop the condition during their ICU stay. These patients have a fourfold higher risk for developing deep vein thrombosis (DVT) compared with ICU patients without renal insufficiency [1-3]. Furthermore, dialysis dependence prior to ICU admission is an independent risk factor for ICU-acquired DVT, potentially related to high levels of fibrinogen, von Willibrand's factor and lipoprotein(a) [4,5], and for treatment with erythropoiesis-stimulating agents [6,7]. Like other critically ill patients, patients with renal insufficiency face an increased risk of DVT because they are typically immobile, undergo invasive procedures, and have large central venous catheters in place for hemodialysis, ultrafiltration or intensive monitoring. At the same time, ICU patients with renal insufficiency are also predisposed to bleeding because of uremic platelet dysfunction, multiple comorbidities, coagulopathies and concomitant treatment with antiplatelet or anticoagulant agents.

Knowledge of the risk factors for, and frequency of, DVT and bleeding may help clinicians to risk-stratify ICU patients with renal insufficiency, and may help guide appropriate thromboprophylaxis. Competing risks of thrombosis and bleeding in these patients poses challenges for thromboprophylaxis. For example, some authorities urge caution in using low-molecular-weight heparin (LMWH) in patients with renal insufficiency [8], and practice guidelines suggest withholding or dose-adjusting LMWH in patients with severe renal insufficiency because of a concern that bioaccumulation may result in bleeding [9]. Most studies of LMWH have systematically excluded patients with renal insufficiency, and few studies have assessed the frequency and determinants of both DVT and bleeding in this challenging high-risk population.

The purpose of the present study was to evaluate the prevalence, incidence and predictors of DVT and major bleeding among critically ill patients with an estimated creatinine clearance <30 ml/min/1.73 m^2^.

## Materials and methods

### Study design

We conducted a multicenter, open-label, prospective cohort study assessing thromboprophylaxis with dalteparin 5,000 IU once daily until ICU discharge or for a maximum of 30 days in critically ill ICU patients with severe renal insufficiency, and we found that dalteparin bioaccumulation did not occur as measured by twice-weekly trough anti-activated factor X (anti-FXa) levels [10]. The purpose of the present report is to estimate the prevalence, incidence and risk factors for venous thromboembolism and bleeding in critically ill patients with severe renal insufficiency.

### Patients

We enrolled adult patients older than 18 years of age, with a body weight >45 kg, with an expected ICU length of stay exceeding 72 hours and with severe renal insufficiency – defined by a creatinine clearance less than 30 ml/min/1.73 m^2 ^based on the Cockroft–Gault formula [11] – or with chronic dialysis. Exclusion criteria were concomitant conditions that increased the risk for bleeding (that is, active bleeding, platelet count <75 × 10^9^/l, International Normalized Ratio or activated partial thromboplastin time >2 times upper limit of normal), a need for therapeutic anticoagulation, life expectancy <14 days, or receipt of palliative care.

Written informed consent for study participation was provided by surrogate decision-makers or patients. This study was approved by the Research Ethics Boards of all participating sites, which are Canadian university-affiliated hospitals with closed mixed medical–surgical ICUs (see Acknowledgements).

### Outcome measures

#### Venous thromboembolism

All patients had bilateral compression lower-limb venous ultrasound within 48 hours of study enrolment and twice weekly thereafter [1] until ICU discharge or 30 days, whichever came first. A standard technique was used to assess venous segments in six locations from the trifurcation to the common femoral vein. This technique, which has high inter-rater reliability in ICU patients [12], was performed by specifically trained study ultrasound technologists and radiologists. DVT was defined by a noncompressible vein segment [13], and was classified as catheter-related if it occurred within 24 hours of placement of a central venous (including dialysis) catheter in the same site. Screening for pulmonary embolism was not conducted routinely; if suspected, pulmonary embolism was diagnosed by standard clinical and diagnostic criteria [14,15].

#### Bleeding

All patients underwent daily bedside clinical assessment using a validated bleeding tool for the ICU population [16]. Bleeding was defined as major if, in the absence of another cause, the patient fulfilled one of three definitions: first, a decrease in hemoglobin by 20 g/l or more in the absence of another cause along with one of either transfusion of more than 2 units packed red cells without a corresponding increase in hemoglobin, a spontaneous decrease in systolic blood pressure by more than 20 mmHg, or an increase in heart rate of 20 beats/min; second, objectively confirmed bleeding at a critical site (for example, retroperitoneal, intracranial); or, finally, bleeding at a wound site requiring an intervention (for example, reoperation). Bleeding that did not satisfy these criteria was defined as minor.

If a major bleed preceded death, a distinction was made between patients dying *with *bleeding (for example, a patient with renal failure and ongoing melena had a hyperkalemic cardiac arrest) and patients dying *due to *bleeding (for example, a patient dying from hypovolemic shock due to massive uncontrolled upper gastrointestinal hemorrhage). Cases fulfilling the former criteria were classified as major bleeding, while the latter criteria were considered to represent fatal bleeding.

#### Anti-activated factor X levels

We measured trough anti-FXa levels twice weekly (Mondays and Thursdays), 20 hours after the prior dalteparin dose, to assess for dalteparin bioaccumulation. The anti-FXa level is approved by laboratory consensus groups to measure the anticoagulant effect of LMWHs [17,18].

The first trough anti-FXa level was measured after a patient had received at least one dose of dalteparin. We also assessed dalteparin pharmacokinetics by measuring anti-FXa levels at 0 hours (baseline), 1 hour, 2 hours, 4 hours, 8 hours, 12 hours, 20 hours, and 24 hours after the prior dalteparin dose on approximately days 3, 10, and 17 after the start of dalteparin. Pharmacokinetic assessments were used to assess the adequacy of anticoagulant effect based on peak anti-FXa levels measured at 2 hours and 4 hours after the prior dalteparin dose.

### Patient management

Patient management was left to the discretion of the ICU team, which was blinded to anti-FXa levels. Patients receiving intermittent hemodialysis continued dalteparin unless a contraindication developed. The catheter patency strategy for patients receiving continuous dialysis was at the discretion of the ICU team, and included no intervention, therapeutic unfractionated heparin or citrate. Research coordinators who prospectively recorded bleeding and DVT outcomes during the study were also unaware of the anti-FXa levels.

### Statistical analysis

For all descriptive analyses, we present categorical data as counts and percentages, and present continuous data as the mean (standard deviation) or median (interquartile range) if data were skewed. The prevalence (at the time of admission to the ICU) and the incidence (during the ICU stay) of DVT and pulmonary embolism are expressed as a percentage and the associated 95% confidence interval (CI). The incidences of major and minor bleeding were expressed as the percentage and 95% CI. From the dalteparin pharmacokinetic data, we present peak anti-FXa levels at 2 hours and 4 hours after drug administration to assess the adequacy of the anticoagulant effect of prophylactic doses of dalteparin.

We performed Cox proportional hazards regression analyses using backwards selection to identify independent risk factors for major bleeding and venous thromboembolism (proximal DVT only, since no incident cases of pulmonary embolism were identified).

In the major bleeding prediction model, the following risk factors were *a priori *independent variables: baseline characteristics (age, Acute Physiology and Chronic Health Evaluation II score [19], surgical versus medical admission, pre-ICU renal status (acute renal failure, acute on chronic renal failure, chronic renal failure not dialysis dependent, dialysis dependent)), and time-dependent predictors (type of dialysis in ICU (continuous or intermittent in preceding 3 days), International Normalized Ratio, activated partial thromboplastin time, platelet count, therapeutic heparin treatment (within preceding 3 days), prophylactic dalteparin (within preceding 3 days), detectable trough anti-FXa level (within preceding 3 days), and any dose of aspirin treatment (within preceding 3 days)).

In the DVT prediction model, the foregoing were *a priori *independent variables (except the detectable trough anti-FXa level) and the following additional risk factors were included: baseline venous thromboembolism risk factors (personal or family history, known thrombophilic disorder, current or recent (in past 5 years) malignancy), and time-dependent predictors (red blood cell transfusion, fresh-frozen plasma transfusion, platelet transfusion, central venous catheter, inotrope or vasopressor dependency, and mechanical ventilation (all within preceding 3 days)).

## Results

### Patient characteristics and management

We enrolled 156 patients with a mean (standard deviation) creatinine clearance of 18.9 (6.5) ml/min within 3.9 (1.6) days of ICU admission. Fifteen patients had prevalent DVT (*n *= 14) or pulmonary embolism (*n *= 1) diagnosed within 48 hours of study enrolment. These 15 patients with prevalent venous thromboembolism (9.6%) were excluded from our assessment of the incidence of DVT and predictors of incident DVT in the ICU, as were three additional patients because of ICU death (*n *= 2) or discharge (*n *= 1) prior to baseline venous ultrasound testing. The baseline characteristics of the 138 patients who received at least one dose of dalteparin and were included in the subsequent analyses are presented in Table 1.

**Table 1:** Characteristics of enrolled patients

Characteristic	Value
Age (years), mean (standard deviation)	68.3 (15.5)
Female, *n *(%)	77 (55.8)
Acute Physiology and Chronic Health Evaluation II score, mean (standard deviation)	27.6 (8.2)
Body mass index (kg/m^2^)^a^	30.7 (8.4)
Surgical admission, *n *(%)	36 (26.1)
Primary admission diagnosis, *n *(%)	
Cardiovascular	15 (10.9)
Pulmonary	33 (23.9)
Gastrointestinal	26 (18.8)
Neurologic	6 (4.3)
Sepsis	31 (22.5)
Metabolic	6 (4.3)
Hematologic	16 (11.6)
Renal	2 (1.4)
Orthopedic	3 (2.2)
Renal classification, *n *(%)	
Acute renal failure	85 (61.6)
Acute on chronic	32 (23.2)
Chronic renal failure	9 (6.5)
Chronic dialysis	12 (8.7)
Creatinine clearance (ml/min/1.73 m^2^), mean (standard deviation)	18.9 (6.5)
Creatinine clearance, *n *(%)	
<10 ml/min/1.73 m^2 ^or chronic dialysis	13 (9.4)
10 to 14.9 ml/min/1.73 m^2^	28 (20.3)
15 to 19.9 ml/min/1.73 m^2^	37 (26.8)
20 to 24.9 ml/min/1.73 m^2^	31 (22.5)
25 to 30 ml/min/1.73 m^2^	29 (21.0)
Intensive care unit length of stay (days), median (interquartile range)	9 (5 to 15)
Management during intensive care unit stay^b^	
Never dialyzed	76 (55.1)
Intermittent hemodialysis only	30 (21.7)
Continuous hemodialysis (includes slow low-efficiency dialysis) only	10 (7.2)
Peritoneal dialysis only	3 (2.2)
Intermittent and continuous hemodialysis	17 (12.3)
Intermittent hemodialysis and peritoneal dialysis	1 (0.7)
Continuous hemodialysis and peritoneal dialysis	0 (0)
Intermittent and continuous hemodialysis, and peritoneal dialysis	1 (0.7)
Intensive care unit mortality, *n *(%)	29 (21.0)

Characteristics of patients enrolled in the DIRECT study, *n *= 138. ^a^Values unavailable for two patients. ^b^Out of 138 patients included in this study, 45% required hemodialysis (intermittent or continuous), or peritoneal dialysis, or a combination.

In total, 121 (87.7%) patients had at least one central venous catheter during their ICU stay (61.6% internal jugular, 1.4% external jugular, 21.0% subclavian, 8.0% brachial and 17.4% femoral). The median duration of dalteparin thromboprophylaxis was 7 days (interquartile range, 4 to 12 days). Dalteparin was held for at least 1 day in 43 patients for a median of 5 days (interquartile range, 1 to 10 days) due to bleeding, potential for bleeding or need for therapeutic anticoagulation. During days in which patients were bleeding or at risk for bleeding, antiembolic stockings were used for a median of 1 day (interquartile range, 1 to 3 days) by 29 (21.0%) patients (12.0% knee-length stockings, 8.0% thigh-length stockings); pneumatic compression devices were used for a median of 3 days (interquartile range, 1 to 8 days) by 11 (8.0%) patients.

### Study outcomes

#### Venous thromboembolism

Seven patients developed DVT (six lower-limb DVT and one upper-limb DVT), for an incidence of 5.1% (95% CI, 2.5 to 10.1). Four DVTs were catheter related. No patient was diagnosed with pulmonary embolism. DVT occurred despite adequate levels of prophylactic anticoagulation: the median peak anti-FXa levels 2 hours and 4 hours after dalteparin administration were 0.29 (interquartile range, 0.19 to 0.39) based on 159 observations in 101 patients, and 0.31 (interquartile range, 0.22 to 0.43) based on 153 observations in 98 patients, respectively.

These mean peak anti-FXa levels achieved with prophylactic-dose dalteparin are consistent with peak prophylactic levels of anticoagulation of 0.20–0.40 IU/ml observed in other hospitalized medical and surgical patients, indicating that our prophylaxis regimen had adequate bioavailability and an appropriate anticoagulant effect [20,21].

#### Bleeding

Ten patients developed major bleeding, and two patients died with bleeding; the incidence was 7.2% (95% CI, 4.0 to 12.8). Twenty-four patients developed minor bleeding, for an incidence of 17.4% (95% CI, 12.0 to 24.6).

#### Predictors of deep vein thrombosis and major bleeding

An increased Acute Physiology and Chronic Health Evaluation II score (hazard ratio for 10-unit difference, 2.25; 95% CI, 1.03 to 4.91) was a risk factor for DVT, as shown in Table 2. Aspirin use (hazard ratio, 6.30; 95% CI, 1.35 to 29.4) and an increased International Normalized Ratio (hazard ratio for 0.5-unit difference, 1.68; 95% CI, 1.07 to 2.66) were risk factors for major bleeding, as shown in Table 3.

**Table 2:** Risk factors for deep vein thrombosis

Putative risk factor	Univariate analysis		Multivariable analysis	
	
	Hazard ratio (95% confidence interval)	*P *value	Hazard ratio (95% confidence interval)	*P *value
Baseline factors				
Age (10-year increase)	0.98 (0.64 to 1.50)	0.91	-	-
Acute Physiology and Chronic Health Evaluation II score (10-point increase)	2.25 (1.03 to 4.91)	0.04	2.25 (1.03 to 4.91)	0.04
Surgical patient	1.96 (0.44 to 8.81)	0.38	-	-
Baseline renal disease classification (with reference to acute renal failure)				
Acute on chronic	1.10 (0.21 to 5.78)	0.91		
Chronic renal failure^a^	-	-		
Chronic hemodialysis^a^	-	-		
Personal or family history of venous thromboembolism^a^	-	-	-	-
Known thrombophilic disorder	6.48 (0.73 to 57.88)	0.09	-	-
Malignancy (current or in past 5 years)^a^	-	-	-	-
Time-dependent factors				
Exposure to continuous hemodialysis^b^	1.56 (0.30 to 8.08)	0.59	-	-
Exposure to intermittent hemodialysis^b^	1.09 (0.21 to 5.67)	0.92	-	-
Exposure to therapeutic-dose unfractionated heparin^b^	-	-	-	-
Exposure to prophylactic-dose unfractionated heparin^b^	0.99 (0.12 to 8.37)	0.99	-	-
Exposure to acetylsalicylic acid^b^	0.58 (0.11 to 2.97)	0.51	-	-
International Normalized Ratio (0.5-unit increase)	1.54 (1.05 to 2.24)	0.03	-	-
Partial thromboplastin time (10-unit increase)	1.08 (0.78 to 1.49)	0.67	-	-
Platelet count (50-unit decrease)	1.09 (0.85 to 1.39)	0.49	-	-
Red blood cell transfusion^a^	1.27 (0.28 to 5.71)	0.75	-	-
Fresh frozen plasma transfusion^a,b^	-	-	-	-
Platelet transfusion^a,b^	-	-	-	-
Central venous catheter placement^b^	-	-	-	-
Exposure to inotropes/vasopressors^b^	0.80 (0.15 to 4.15)	0.79	-	-
Exposure to mechanical ventilation^b^	-	-	-	-
Peak anti-FXa levels (most recent measurement prior to DVT diagnosis)^c^	0.04 (0.0 to 97.4)	0.43	-	-

^a^No patient with deep venous thrombosis (DVT) had this putative risk factor. ^b^Within 3 days prior to study enrollment. ^c^Three out of seven patients with DVT did not have a peak anti-activated factor X (anti-FXa) level measured on the day of diagnosis of DVT; for this reason, the peak anti-FXa level was not included in the multivariable analysis.

**Table 3:** Risk factors for major bleeding

Putative risk factor	Univariate analysis		Multivariable analysis	
	
	Hazard ratio (95% confidence interval)	*P *value	Hazard ratio (95% confidence interval)	*P *value
Baseline factors				
Age (10-year increase)	0.82 (0.58 to 1.15)	0.25	-	-
Acute Physiology and Chronic Health Evaluation II score (10-point increase)	0.97 (0.49 to 1.93)	0.93	-	-
Surgical patient	1.76 (0.50 to 6.26)	0.38	-	-
Baseline renal disease classification (with reference to acute renal failure)				
Acute on chronic	0.92 (0.18 to 4.56)	0.91		
Chronic renal failure^a^	-	-		
Chronic hemodialysis	3.59 (0.71 to 18.15)	0.12		
Time-dependent factors				
Exposure to continuous hemodialysis^b^	2.17 (0.55 to 8.51)	0.27	-	-
Exposure to intermittent hemodialysis^b^	1.25 (0.32 to 4.85)	0.75	-	-
Exposure to therapeutic-dose unfractionated heparin^b^	2.87 (0.59 to 13.97)	0.19	-	-
Exposure to prophylactic-dose unfractionated heparin^b^	1.10 (0.14 to 8.80)	0.93	-	-
Exposure to acetylsalicylic acid^b^	3.62 (0.93 to 14.11)	0.06	6.30 (1.35 to 29.35)	0.02
Detectable trough anti-activated factor X level (≥ 0.01 IU/ml)	0.76 (0.15 to 3.81)	0.74	-	-
International Normalized Ratio (0.5-unit increase)	1.44 (0.95 to 2.17)	0.09	1.68 (1.07 to 2.66)	0.03
Partial thromboplastin time (10-unit increase)	1.13 (0.99 to 1.29)	0.06	-	-
Platelet count (50-unit decrease)	1.19 (0.94 to 1.51)	0.16	-	-

^a^No patient with major bleeding had this putative risk factor. ^b^Within 3 days prior to study enrollment.

## Discussion

There are two principal findings of this study of thromboprophylaxis with dalteparin 5,000 IU once daily in critically ill patients with severe renal insufficiency. First, the incidence of DVT and major bleeding are considerable – at 5% and 7%, respectively – consistent with outcome rates in other studies involving critically ill patients receiving different methods of thromboprophylaxis. Second, our assessment of risk factors for DVT and major bleeding suggests that these outcomes were a consequence of comorbidities. We did not find that an inadequate anticoagulant effect contributed to DVT, and we did not find that an excessive anticoagulant effect contributed to major bleeding.

The 5% incidence of proximal DVT we observed is consistent with other studies in critically ill patients receiving thromboprophylaxis. In a study of 261 critically ill patients (with or without renal insufficiency) who received protocol-directed thromboprophylaxis with unfractionated heparin 5,000 IU twice daily, the ICU incidence of proximal DVT was 9.6% (95% CI, 6.3 to 13.8) based on twice-weekly venous ultrasound screening that was the same screening approach [1] as used in the present study. Furthermore, in the present study, DVT occurred despite adequate levels of prophylactic anticoagulation, based on peak anti-FXa levels of approximately 0.30 IU/ml. Although anti-FXa levels were missing for three of the seven patients on the day DVT was diagnosed, previous anti-FXa levels for each of these patients were within the range observed with adequate anticoagulant prophylaxis regimens (0.20–0.40 IU/ml). Taken together, these findings suggest that ICU patients are a moderately high-risk group for developing DVT, and that anticoagulant thromboprophylaxis does not completely mitigate the risk for DVT.

Our finding that an increase in the Acute Physiology and Chronic Health Evaluation II score conferred an increased risk for DVT is plausible given that this score reflects the presence and severity of acute and chronic morbidities. Furthermore, most critically ill patients are immobile, have central venous catheters in place and undergo numerous other procedures, all of which factors can increase the risk for DVT. Although we did not find that conventional DVT risk factors such as thrombophilia or personal or family history of thrombosis conferred an increased risk for DVT, this may reflect the low prevalence of thrombophilia and a relatively underpowered regression analysis.

Bleeding rates in ICU patients reported in the literature reflect the objectives and case mix of individual studies. The 7% incidence of major bleeding we observed reflects the exclusion of patients who were bleeding or at high risk of bleeding at the time of screening, as is typical for heparin thromboprophylaxis studies. In a previous series of 19 medical–surgical ICU patients without renal insufficiency receiving dalteparin prophylaxis, only one patient had clinically important bleeding [22]. On the other hand, in a study to estimate bleeding rates in an unselected cohort of 100 consecutive ICU patients who received unfractionated heparin 5,000 IU twice daily, the incidence of major bleeding was 20% and most bleeding episodes were minor, recurrent and wound or procedure-related [16]. In the present study, only 25 (5.6%) of the 480 bleeding events were major.

We found that acquired coagulopathy (for example, increased International Normalized Ratio) and concomitant use of drugs affecting hemostasis (for example, prior aspirin use) were independent risk factors for major bleeding, which is biologically plausible and consistent with other studies assessing bleeding risk factors in patients who are receiving anticoagulants. As all patients received dalteparin, it is not possible to make definitive inferences about the effect of LMWH use on bleeding risk. We found, however, that a detectable trough anticoagulant effect from dalteparin thromboprophylaxis, which may be considered a surrogate marker for excessive anticoagulation, was not associated with an increased risk for bleeding (odds ratio, 0.76; 95% CI, 0.15 to 3.81). The latter finding is consistent with a systematic review of 11 studies of noncritically end-stage dialysis-dependent renal failure patients that demonstrated no association between bleeding or extracorporeal circuit thrombosis and LMWH or unfractionated heparin [23].

Our conclusions may be affected by the definitions of bleeding. While there are multiple definitions of major and minor bleeding in use, setting-specific reporting is necessary. Koreth and colleagues identified over 16 published bleeding scales, none of which were designed to capture clinically important bleeding in critically ill patients [24]. Even among existing tools, no standardized definitions exist. A systematic review by Raskob and colleagues identified 18 definitions of severe bleeding among 62 studies of hip surgery thromboprophylaxis [25]. In the present study, we used a clinically useful bleeding measurement tool developed and validated in the ICU population [16].

Limitations of the present study include the sample size. Although this is the largest cohort study of ICU patients with renal insufficiency receiving LMWH thromboprophylaxis, the number of patients is a major determinant of the power of regression analysis to identify risk factors for a prespecified outcome. Previous studies involving critically ill patients [26] or medical patients [27] were small and did not assess risk factors for adverse clinical outcomes using multivariate analysis. Also, the importance of certain risk factors depends on the prevalence of the risk factor in the population studied. We performed screening venous ultrasound testing of the lower but not the upper extremities, so we may have underestimated the DVT rate overall. We did not identify central venous catheterization [28] as an independent predictor in our multivariate regression, in part because almost all patients had central venous catheters. Indeed, four of seven incident DVTs observed in this study were catheter-related. Merrer and colleagues, however – in a randomized trial of femoral catheter versus subclavian central venous catheter insertion – found the catheter site to be an important determinant for DVT risk, as femoral catheters conferred a higher risk for DVT [29].

Comparing DVT risk factor analyses across studies is methodologically challenging in this field, owing to differences in case mix, consideration of different candidate risk factors, variable approaches to the diagnosis of DVT (including cases comprehensively identified by screening versus a minority of DVTs identified by physical examination), the different thromboprophylaxis agents used, variable DVT location (proximal and/or distal, lower-limb DVTs versus all DVTs), reliance on univariate analyses rather than multivariate analyses, and typically unreported events and exposures over the ICU course such that risk factors include patients' baseline characteristics only.

Strengths of the present study include daily screening for consecutive eligible patients to be enrolled, thereby minimizing the potential for selection bias. The ICU team and research coordinators made comprehensive daily bleeding assessments with a validated tool [16] and were blinded to the anti-FXa levels. DVT was identified by twice-weekly screening venous ultrasound, which has been protocolized in previous studies [1,30] and has been shown to be reliable [12]. These design features made biased interpretation of clinical outcomes unlikely in this open-label study. We focused on independent predictors of bleeding using multivariate analysis. Enrolment of patients with a range of renal insufficiency including dialysis dependence, from multiple centers, enhances the generalizability of our findings.

## Conclusion

In summary, in the present multicenter observational study of ICU patients with renal insufficiency, we observed that the incidence of DVT and major bleeding is considerable. These events, however, appear related to patient comorbidities rather than an inadequate or excessive anticoagulant effect from thromboprophylaxis with dalteparin.

## Key messages

• Critically ill patients with renal insufficiency are predisposed to both DVT and bleeding.

• In this multicenter study, ICU patients with estimated creatinine clearance <30 ml/min receiving dalteparin 5,000 IU daily as thromboprophylaxis did not develop dalteparin bioaccumulation, as measured by twice-weekly trough anti-FXa levels.

• DVT developed in 5.1% of patients. The only independent DVT risk factor was a high baseline Acute Physiology and Chronic Health Evaluation II score (hazard ratio for 10-point increase, 2.25).

• Major bleeding developed in 7.2% of patients. The independent risk factors for major bleeding were aspirin use (hazard ratio, 6.30) and a high international normalized ratio (hazard ratio for 0.5-unit increase, 1.68).

• Although DVT and major bleeding occurred during critical illness among patients with severe renal insufficiency, these events were not associated with an inadequate or an excessive dalteparin anticoagulant effect, respectively.

## Abbreviations

Anti-Fxa = anti-acitvated factor X; CI = confidence interval; DVT = deep vein thrombosis; ICU = intensive care unit; LMWH = low-molecular-weight heparin.

## Competing interests

The present study was an investigator-initiated study of the Canadian Critical Care Trials Group, with methods-center funding from the Canadian Institutes of Health Research and an unrestricted arms-length grant-in-aid from Pfizer (Montreal, Canada), which supplied the dalteparin. Neither sponsor had any role in the design, execution, analysis, interpretation or publication of this study.

## Authors' contributions

DC, JD, MM, GG, CR and MC obtained funding. JD, DC, MM, GG, JG, YS, MA, PH, CR, WG and MC were responsible for conception and design of the study. DC, MM, NZ, JG, YS, MA, RF, PH, GP, JF, AF and TK performed the data collection. DC, JD, MM, GG and DH-A performed the statistical analysis. DC, JD, MM, GG, CR, DH-A and MC drafted the article. NZ, JG, YS, MA, RF, PH, GP, JF, AF, TK and WG critically revised the article. DC was the guarantor.
